# Night-time warming and prey availability interact to influence physiology in prairie lizards (*Sceloporus consobrinus*)

**DOI:** 10.1242/jeb.250737

**Published:** 2025-08-14

**Authors:** Allison R. Litmer, Morgan K. Pelley, Steven J. Beaupre

**Affiliations:** ^1^ The Ohio State University, 1314 Kinnear Road, Columbus, OH 43212, USA; ^2^ University of Arkansas, 650 W. Dickson Street, Fayetteville, AR 72701, USA

**Keywords:** Bioenergetics, Climate change, Digestion, Foraging, Reptile, Temperature cycle

## Abstract

Among ectotherms, temperature and food availability are drivers of individual and population characteristics. Increasing night-time temperatures due to climate change can increase energetic demands, potentially amplified or mitigated by prey consumption. Few studies examine night-time temperatures specifically, and consequences of co-occurring changes in the abiotic and biotic environment. Thus, we examined interactions between warming night-time temperatures and prey availability (as a proxy of foraging time and effectiveness), and how they affect food consumption, digestive passage time, metabolizable energy intake (MEI), assimilated energy, and fecal and urate production in a model organism, prairie lizards (*Sceloporus consobrinus*). Three temperature treatments (current conditions, +2°C night-time increase and +4°C night-time increase) and three levels of prey availability (low, moderate and high) were used to examine multiple scenarios. Prey and warming interacted in non-linear and unpredictable ways to influence food consumption, passage time and MEI. For example, lizards consumed the most food under 2°C warming yet excreted a high proportion of energy resulting in low MEI in comparison to current temperatures. The combined effects of prey availability and night-time warming will be missed by studies only considering single treatment effects. Moreover, physiological processes can have different thermal sensitivities to night-time temperatures, which can vary in unpredictable ways with combined biotic changes. Complex interactions between thermal sensitivities and multiple concurrent environmental changes may be common among ectotherms, complicating our understanding of responses to a shifting climate.

## Teaser

**Summary:** Warming night-time temperatures and changing food availability show interactive effects on energetics and physiology in prairie lizards (*Sceloporus consobrinus*), highlighting challenges in predicting climate change impacts.

## INTRODUCTION

While understanding the interaction between organisms and climate is not a new topic (Porter and Gates, 1969; Parmesan, 2006), it has become of increasing focus in response to projections of anthropogenic climate change (Williams et al., 2009; Heino et al., 2009). Physiology, which can be influenced by the environment for ectotherms (Huey, 1982; Ohlberger, 2013; Sokolova, 2021), plays an important role in determining individual fitness and life history (Congdon et al., 1982; Dunham et al., 1989). Therefore, studies examining interactions among inter-dependent physiological processes and external factors are critical for understanding the influence of climate on organisms (Urban et al., 2016). Environmental change can induce biotic change (Andrén et al., 1985; Haile, 2020), such as altered prey abundance, predation and disease, which could have interactive effects on organisms (Dunham et al., 1989; Coors and De Meester, 2008). Consideration of how co-occurring abiotic and biotic changes can influence physiology is needed for enhancing ecological theory on drivers of population change and informing climate change studies and predictions (Davis et al., 1998; Beaupre, 2002; Poloczanska et al., 2008; Williams and Middleton, 2008; Huey and Kingsolver, 2019).

Persistence and life history are known to vary in response to energetic trade-offs, induced by both the abiotic and biotic environment (Dunham et al., 1989; Thunell et al., 2023). The environment can impose constraints on behaviors that have the potential to be favorable under different circumstances, if all other conditions (e.g. predators, space, food availability, etc.) were equal. For example, temperature restrictions can prevent organisms from being active, which can reduce foraging time or affect mate search behavior (Chong and Lee, 2009; Oyugi et al., 2012; Pilakouta and Baillet, 2022; Plasman et al., 2025). Reductions in rainfall or humidity can also reduce activity in the interest of preventing dehydration, at the expense of engaging in other beneficial tasks (Berger et al., 2014; Pintor et al., 2016; Dubos et al., 2020). If such abiotic changes occur in tandem with biotic changes, such as altered prey availability, effects could interact to have beneficial or negative consequences (Gilman et al., 2010; Vucic-Pestic et al., 2011; Dell et al., 2014). However, most studies examine singular environmental attributes and lack the ability to mechanistically assess how changes in multiple parameters can interact to influence ectotherms. Therefore, to enhance our understanding of the underlying mechanisms of identified ecological phenomena, we need to assess the direct effects of co-occurring changes in the abiotic and biotic environment.

For many ectotherms, temperature and food are primary drivers of performance and fitness. Various studies have examined the influence of temperature change on prey acquisition and consumption, with particular focus on population and community ecology (e.g. Post and Pederson, 2008; Brodie et al., 2012; Dell et al., 2014; Brewster et al., 2020). With respect to ectotherms, temperature can strongly influence behavior, duration of activity and rates of physiological processes (Grant and Dunham, 1988; Gunderson and Leal, 2015; McMunn and Pepi, 2022). Warmer temperatures have been found to increase rates of food consumption and prey attacks and decrease handling time in ectotherms (Persson, 1986; Hewitt and Duncan, 2001; Niu et al., 2003; Haubrock et al., 2020). Increases in food consumption have been predicted, and documented, to have cascading effects on communities through changes in abundance and behavior at different trophic levels (Rodríguez-Castañeda, 2013; Veselý et al., 2019; Durant et al., 2019). While it is known that functional relationships and physiological processes can vary in response to temperature, less attention has been paid to the influence of interacting effects of temperature and food on individuals. Furthermore, the vast majority of thermal studies rely upon data acquired when animals experience stable temperature regimes, which may not be reflective of daily temperature cycles in nature (Kingsolver et al., 2015; Dillon et al., 2016; Litmer and Beaupre, 2024). Therefore, a nuanced understanding of thermal sensitivity under daily cycling regimes, and how such sensitivity can vary with combined abiotic change, would be beneficial to expanding ecological theory.

Energy available for allocation to growth, storage, maintenance and reproduction is primarily dictated by the amount of food animals can consume (Congdon et al., 1982). However, food consumption is often temperature dependent for ectotherms and can increase, stabilize or decline as temperatures warm (Huey, 1982; Beaupre et al., 1993b; Angilletta, 2001b; Volkoff and Rønnestad, 2020). Warmer body temperatures (*T*_b_) also increase metabolic rate (Beaupre et al., 1993a; Angilletta, 2001a; Hölker, 2003), while decreasing digestive passage time (time to pass a single food item from consumption to excretion; Waldschmidt et al., 1986; Plasman et al., 2019). Subsequently, higher metabolic rates can result in the need for greater food consumption to meet energetic demands. Therefore, if warming temperatures are paired with decreased food availability, ectotherms could experience critical declines in energy budgets (Beaupre, 1995), as energetic costs associated with maintenance and food acquisition could increase without sufficient prey. Such changes in individual energy budgets with temperature could have subsequent effects on populations. Even when ectotherms maintain current *T*_b_, prey decreases could result in smaller energy budgets, potentially altering reproduction, life history and persistence (Brewster et al., 2020).

When considering combined effects of temperature and prey availability, a variety of scenarios are possible. Researchers often study the effects of changing daily *T*_b_, or temperatures exceeding critical thermal limits (Deutsch et al., 2008; Nascimento et al., 2022). However, many ectotherms actively regulate *T*_b_ during the day through behavioral and physiological mechanisms, as the sun and habitat structures can provide thermal heterogeneity (Huey and Slatkin, 1976; Ouedraogo et al., 2004; Leith et al., 2024). At night, in the absence of incoming solar radiation, the thermal landscape becomes more homogeneous, and many diurnally active ectotherms cease activity and find retreats. When activity ceases at night, an ectotherm's *T*_b_ reaches equilibrium with the surrounding habitat (Porter and Gates, 1969). Therefore, if environmental temperatures change, night-time temperatures may have a greater influence than daytime temperatures when thermoregulation is possible. Additionally, some studies indicate that night-time temperatures are warming more rapidly than daytime temperatures as a result of climate change (Vose et al., 2005; Davy et al., 2017). Despite the potential importance of warming night-time temperatures, little information exists on whether and how changing night-time temperatures influence ectotherm fitness and performance. However, it is possible that a third to half of an animal's life is spent under night-time temperature regimes; therefore, they are likely to contribute heavily to regulating temperature-dependent processes in ectotherms. To better understand thermal sensitivity to daily temperature cycles, ecological factors regulating performance and threats of climate change, empirical data that examine combined effects of night-time warming and changes in the biotic environment are needed.

Lizards have been the focus of studies of thermal biology and life history for decades (e.g. Porter and Gates, 1969; Huey and Slatkin, 1976; Beaupre et al., 1993b; Sears, 2005; Hao et al., 2021). Lizards in the genus *Sceloporus* are particularly useful for studying the influence of environmental attributes, including night-time temperature, on ectotherms because they have a known sensitivity to temperature and are widely distributed with variable life history (Tinkle and Ballinger, 1972; Beaupre et al., 1993b; Niewiarowski, 1995; Angilletta, 2001b; Buckley, 2008). Furthermore, *Sceloporus* lizards are diurnal and insectivorous, which is comparable to many other ectotherms, and data derived from *Sceloporus* research can inform future theories relatable to other ectotherms.

The objectives of the current study were to quantify and demonstrate the effects of warming night-time temperatures and variable prey availability on six physiological traits in prairie lizards (*Sceloporus consobrinus*). Specifically, rates of food consumption, digestive passage time, metabolizable energy intake (MEI; see Materials and Methods), assimilated energy (AE; see Materials and Methods), fecal production and urate production were measured in a full factorial design under nine scenarios of night-time warming and prey availability. We were particularly interested in the effects of prolonged periods exposed to different levels of prey availability in conjunction with warmer night-time temperatures, as opposed to just the effects of food consumption on physiology. Temperature treatments were designed to (1) mimic a current temperature regime (e.g. typical of NW Arkansas), (2) mimic a 2°C night-time warming scenario, and (3) mimic a 4°C night-time warming scenario, as forecasted by the Intergovernmental Panel on Climate Change (IPCC). Prey availability treatments represented low, moderate and high prey, based on quantified food consumption rates for *S. consobrinus*. The physiological variables of interest were selected because they directly influence energy budgets which influence fitness and likely vary in response to prey and temperature. We hypothesized that lizards would increase consumption, MEI, AE, fecal production and urate production with warming night-time temperatures, while passage time (time from ingestion to egestion) would decrease, as ectotherm physiology often has higher rates at warmer temperatures. We also hypothesized that lizards would proportionally increase food consumption and energy budgets in relation to prey availability. Lastly, we hypothesized that warm temperature regimes (that increase metabolism and nitrogen turnover) paired with low prey would produce a constraint on metabolizable energy intake. Our findings herein identified mechanistic and interactive relationships between thermal sensitivity to night-time warming and prey availability for multiple physiological traits. Physiological traits varied in response to changing night-time temperatures and prey availability, demonstrating the complexity of such changes, which is likely important to regulating natural systems.

## MATERIALS AND METHODS

### Study organism and field temperature measurements

Adult *Sceloporus consobrinus* Baird & Girard 1854, ranging from 53 to 73 mm snout–vent length (SVL) and 5.3 to 12.0 g mass, were captured by hand or with a lizard loop in northwest Arkansas, USA, from 2021 to 2022. The habitat where lizards were located consisted of pine-hardwood and mixed-oak forests with high rock abundance and openings for the sun to penetrate to the forest floor. Sex was determined via the presence (male) or absence (female) of post-anal scales. Gravid females were not used for the current study. Lizards were immediately brought to the University of Arkansas and individually housed in 37.851 l tanks with a natural sand substrate, a heat lamp and a hide box, with water provided *ad libitum*. Lizards were fed a diet of crickets, were misted with water every other day, and supplemented with vitamin D by dusting every 2 weeks.

To determine current *T*_b_ profiles of *S. consobrinus* for use in lab trials, field data were collected. To determine daytime *T*_b_ of active lizards, measurements were made in 2020 and 2021 by inserting a fine wire thermocouple (Type K, Omega Engineering, Norwalk, CT, USA) into the cloaca of active lizards captured within 2 min of being located. To inform lab trials, daytime *T*_b_ was averaged across all measurements taken. To determine night-time retreat temperature, 45 iButton temperature loggers, logging temperature every 15 min, were placed in lizard retreats (primarily rock crevices) at field sites in 2021 for three consecutive nights in March, April, May, June, July and August. To inform lab trials of night-time *T*_b_, all night-time retreat temperatures (from 17:00 h to 10:00 h based on observed activity times) were averaged. It was assumed that at night, lizard *T*_b_ was in equilibrium with retreat temperature (Porter and Gates, 1969). Necessary permits were acquired from the Institutional Animal Care and Use Committee at the University of Arkansas (#19080) and Arkansas Game and Fish Commission (#050120211).

### Night-time warming treatments

Three temperature treatments were implemented, one representing current conditions and two scenarios reflecting climate change forecasts. The IPCC created a goal of maintaining a 2°C or less global temperature increase to reduce catastrophic environmental and economic consequences (IPCC, 2019). However, it is predicted that the world is on a trajectory to a global mean increase of 3–5°C, or at least exceeding +2°C temporarily before dropping again, upon future reductions in emissions (Knutti et al., 2015; Dunlop and Spratt, 2018). Here, we attempted to bound the problem, recognizing that a 4°C increase in global temperature may not result in a 4°C increase in retreat temperature. We implemented two trials to approximate climate predictions, one consisting of a 2°C night-time temperature increase and another consisting of a 4°C night-time temperature increase. Daytime *T*_b_ in every trial reflected the average *T*_b_ of active lizards under current conditions. Therefore, the three temperature treatments all had a daytime temperature of 32.4°C (based on field data), and treatment one (current conditions) had a night-time temperature of 20.4°C (based on field data), treatment two (+2°C) had a night-time temperature of 22.4°C and treatment three (+4°C) had a night-time temperature of 24.4°C. All trials followed a cycle of 8 h at the daytime temperature and 16 h at the night-time temperature.

### Prey availability treatments

Three prey availability treatments (high, medium and low) were implemented in a full factorial design across temperature treatments. To determine prey availability treatments, a preliminary lab study was conducted at 33°C (temperature maintained with an environmental chamber, ±1.0°C) to determine the amount of food a lizard would voluntarily eat when near their average daytime *T*_b_. For the preliminary study, lizards (*n*=9) were placed in plastic containers (41.9 cm×33 cm×16.8 cm) with a hide box and water bowl and acclimated for 5 days to 33°C. Lizards were fasted for the last 3 days of acclimation to ensure the gut was empty. To quantify voluntary consumption rate, lizards were offered Fluker's 2- and 3-week-old crickets weighed to the nearest 0.1 mg, until voluntary feeding ceased. Once lizards stopped eating, the final cricket was left in the tank for 2 h before removal to ensure satiation. Fasted lizards, on average, consumed 0.49±0.2 g (mean±s.d.) wet mass of crickets at 33°C, which was used to determine the high prey availability treatment. The minimum consumed by lizards in the preliminary study was 0.16 g wet mass, which was used to determine the low prey availability treatment.

Based on the results of the voluntary food consumption rates, lizards in the high, moderate and low prey availability treatments were offered 0.45±0.025, 0.3±0.025 and 0.15±0.025 g wet mass daily. The high prey availability treatment was slightly lower than the laboratory observed maximum consumption, because the preliminary trial was based on fasted animals, whereas in the actual prey availability treatments for the current study, lizards were fed daily. Additionally, the average *T*_b_ in the preliminary trial was higher than that in the actual night-time warming treatments. The low prey availability treatment was determined by rounding to the nearest whole number the lowest observed consumption in the preliminary trial. The low value was selected knowing that lizards would be offered food daily and with the objective of preventing starvation or decreased body condition. The moderate prey availability treatment was selected to bisect the high and low treatments. Lizards were randomly assigned to nine independent treatments, with males and females as evenly divided as possible to randomize the potential effect of sex. Treatments consisted of: (1) current conditions (32.4°C day, 20.4°C night) with high (*n*=10 lizards; 7 female, 3 male), moderate (*n*=10 lizards; 6 female, 4 male) and low (*n*=8 lizards; 5 female, 3 male) food availability, +2°C conditions (32.4°C day, 22.4°C night) with high (*n*=10 lizards; 6 female, 4 male), moderate (*n*=11 lizards; 5 female, 6 male) and low (*n*=8 lizards; 4 female, 4 male) food availability, and +4°C conditions (34.4°C day, 24.4°C night) with high (*n*=10 lizards; 4 female, 6 male), moderate (*n*=9 lizards; 4 female, 5 male) and low (*n*=10 lizards; 5 female, 5 male) food availability.

### Feeding trials

During lab trials, lizards were housed in plastic tanks (41.9 cm×33 cm×16.8 cm), lined with butcher paper, with a hide box and water provided *ad libitum*. Lizards were acclimated to their respective temperature treatments 5 days prior to the onset of trials. At the beginning of the acclimation period, lizards were fed a single meal of multiple crickets which they ate until satiation to allow for digestion under their treatment temperatures, and then they were fasted for 3 days to ensure the gut was empty. Temperature was maintained using a walk-in environmental chamber (±0.5°C). During trials, lizards were fed Fluker's 2- and 3-week-old crickets, weighed to the nearest 0.1 mg every morning, and allotted ∼2 h for consumption. Any uneaten food remaining after this time was removed, reweighed and subtracted from the wet mass offered to determine grams wet mass consumed by a lizard.

To begin lab trials, a single cricket was injected with a marker, which was a slurry made by mixing inert UV-fluorescent powder (Scientific Marking Materials Inc., Seattle, WA, USA) with water (Beaupre et al., 1993a,b; Beaupre and Zaidan, 2012). The fluorescent powder associates with feces (Beaupre et al., 1993b), and does not influence edibility of the cricket, so consumption is voluntary. The time lizards consumed the first marker was noted, and then tanks were monitored every 2 h for feces. After the first marker was excreted, typically 10+ days were allotted to feeding the lizards and collecting feces and urates, to adequately measure consumption and collect samples for calorimetry. Following this period, a second marked food item was fed to the lizards, and the tanks were monitored again every 2–4 h until the first appearance of the second marker in feces. Trials were considered complete at the first appearance of the second marked food item in feces. Feces and urates were collected, and consumption measured, during trials in between the first appearance of the first marker and the first appearance of the second marker. All feces and urates were separated for each lizard, frozen and freeze dried.

### Data collection

To determine food consumption (kJ), the wet mass of 20 crickets was measured to the nearest 0.1 mg. Crickets were then freeze dried and reweighed to determine the percentage of water. The percentage of water in crickets was used to convert wet mass consumed by lizards into dry mass consumed. The energy density of crickets was then determined by homogenizing the 20 freeze-dried crickets, which were analyzed in triplicate using bomb calorimetry (Parr Semimicro Calorimeter 6725, Moline, IL, USA). The results were averaged and used to convert dry mass consumed into kilojoules. Digestive passage time is a measurement of the time it takes to pass a food item from consumption to excretion. Passage time was calculated as the time from consumption of the marked cricket to the first appearance of the marker in feces.

Fecal samples were collected throughout the trial and were pooled for each individual lizard. Feces were then homogenized, weighed and analyzed via bomb calorimetry (Parr Semimicro Calorimeter) to determine the energy density. The same methods used to determine fecal production were used to determine urate production. Urate samples collected throughout a trial were pooled for each individual lizard, homogenized, weighed and analyzed via bomb calorimetry to determine the energy density.

Assimilated energy represents digestible energy, and was calculated using the formula:
(1)

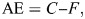
where *C* is energy consumed (kJ) and *F* is energy lost as feces (kJ), and was calculated for the entire trial period. Metabolizable energy intake measures the maximum potential energy to be allocated towards growth, maintenance, storage and reproduction, and was calculated using the formula:
(2)

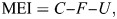
where *C* is energy consumed (kJ), *F* is energy lost as feces (kJ) and *U* is energy lost as uric acid (kJ), and was calculated for the entire trial period.

### Statistical analysis

To determine whether food consumption (kilojoules consumed) and passage time differed among prey availability and night-time warming treatments (predictors), analyses of covariance (ANCOVA) were run with lizard SVL as a covariate, main effects and interaction terms for all combinations of predictors. To determine whether there was an influence of night-time warming and prey treatment on fecal production, urate production, MEI and AE, ANCOVA were run with food consumed in kilojoules as a covariate and interaction terms for all combinations of predictors. Prey treatment reflects the condition of prolonged availability of prey whereas food consumption is a continuous variable of how much lizards consumed in trials. *Post hoc* Tukey tests were performed to determine differences among night-time warming treatments when analyses were significant. The residuals from all analyses were assessed for meeting the assumptions of parametric statistics. A Type I error probability of α=0.05 was adopted for all statistical procedures. All analyses were run in R (version 4.1.3; http://www.R-project.org/). ANCOVA were conducted using the package ‘car’ (Fox and Weisberg, 2019) and adjusted means and *post hoc* analyses were conducted using the package ‘emmeans’ (https://CRAN.R-project.org/package=emmeans). All means are presented ±s.d. unless otherwise indicated.

## RESULTS

While residuals for energetic metrics deviated slightly from normality, all followed a unimodal hump-shaped distribution and were deemed suitable for the robust procedures of ANCOVA (Blair, 1981). The prey availability treatments were successful in achieving variation in food consumption for all temperature treatments, ranging in consumption from 2.95 to 30.34 kJ (mean±s.d. 12.64±1.10 kJ, *n*=30) for the current condition treatment, 5.13 to 26.84 kJ (mean±s.d. 16.52±1.11 kJ, *n*=29) for the +2°C treatment, and 3.36 to 26.40 kJ (mean±s.d. 12.46±1.03 kJ, *n*=29) for the +4°C treatment. For high food availability treatments, lizards rarely consumed all food offered and regularly ate until satiation, while lizards commonly ate all food offered in the low prey treatment.

### Food consumption

**Fig. 1.**
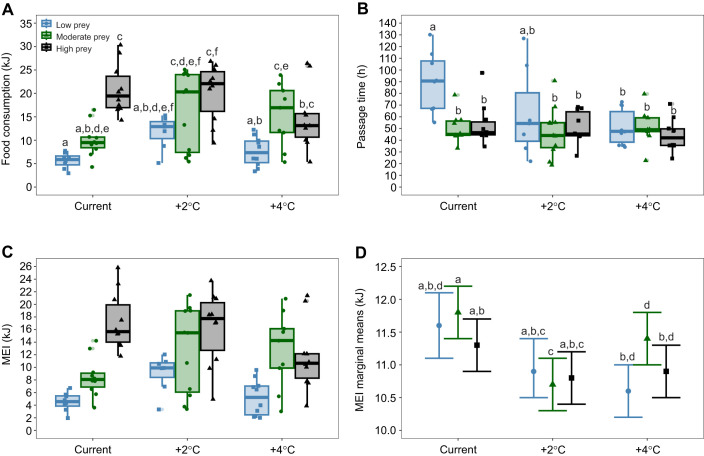
**Food consumption, passage time and metabolizable energy intake (MEI) in prairie lizards (*Sceloporus consobrinus*) subjected to night-time warming and prey availability treatments.** Night-time warming and prey availability interacted to influence food consumption (A), passage time (B) and MEI (C,D) in prairie lizards. (C) Raw data points for MEI and (D) marginal means adjusted for the effects of food consumption from ANCOVA analyses when lizards experienced low, moderate or high prey availability and temperature treatments reflecting current conditions, or night-time warming by 2°C and 4°C. Boxplots show median, error bars represent 95% confidence intervals and letters above each boxplot or mean indicate significant differences.

The energy density of crickets was 23.2±0.28 kJ g^−1^ dry mass and the water content of crickets was ∼75%. ANCOVA revealed that the covariate of SVL was not a significant covariate for food consumption, so the analysis was reduced to two-way ANOVA. Night-time warming (ANOVA *P*<0.0001, *F*=155.1) and prey availability treatment (ANOVA *P*<0.0001, *F*=21.4) significantly influenced kilojoules consumed by lizards (Fig. 1A). Night-time warming and prey availability showed a significant interaction effect on kilojoules of food consumed by lizards (ANOVA *P*=0.0275, *F*=2.9). Specifically, food consumption was significantly lower for lizards under current conditions with low prey availability in comparison to lizards under moderate prey availability with 2°C and 4°C night-time warming, or high prey under any temperature regime (Table 1). Additionally, lizards experiencing 2°C night-time warming and low prey availability had significantly lower food consumption than lizards under current conditions and high prey availability. Lizards experiencing 4°C night-time warming and low prey availability consumed significantly less food than lizards with moderate prey availability under 2°C and 4°C night-time warming, and lizards with high prey under 2°C warming and current conditions. Lastly, lizards under current conditions with moderate prey availability consumed significantly less food than lizards with high prey under current conditions or 2°C warming.

**Table 1.** Night-time warming and prey availability showed an interaction effect with food consumption (kJ) in prairie lizards (*Sceloporus consobrinus*)

Food availability	Temperature treatment	Low food			Moderate food			High food		
		Current	+2°C	+4°C	Current	+2°C	+4°C	Current	+2°C	+4°C
Low	Current	1								
	+2°C	*T*=−6.0807 *P*=0.4705	1							
	+4°C	*T*=−1.9473 *P*=0.9980	*T*=−4.1334 *P*=0.8472	1						
Moderate	Current	*T*=−4.4231 *P*=0.7569	*T=*1.6576 *P=*0.9995	*T=*−2.4758 *P=*0.9851	1					
	+2°C	*T*=−10.5843 *P*=0.0031*	*T=*−4.5036 *P=*0.7580	*T=*−8.6370 *P=*0.0176*	*T=*−6.1612 *P=*0.2302	1				
	+4°C	*T*=−9.7504 *P*=0.0150*	*T=*−3.6697 *P=*0.9249	*T=*−7.8031 *P=*0.0176*	*T=*−5.3273 *P=*0.4890	*T=*−0.8339 *P=*1.000	1			
High	Current	*T*=15.2111 *P*<0.0001*	*T=*9.1304 *P=*0.0337*	*T=*13.2638 *P<*0.0001*	*T=*10.7880 *P=*0.0013*	*T=*4.6268 *P=*0.6111	*T=*5.4607 *P=*0.4545	1		
	+2°C	*T*=14.6057 *P*<0.0001*	*T*=8.5249 *P*=0.0613	*T=*12.6584 *P*=0.0001*	*T=*10.1826 *P=*0.0031*	*T=*4.0124 *P=*0.7697	*T=*4.8553 *P=*0.6137	*T=*0.6054 *P=*1.000	1	
	+4°C	*T=*9.0825 *P=*0.0245*	*T*=3.0018 *P*=0.9730	*T=*7.1352 *P=*0.1118	*T=*4.6594 *P=*0.6317	*T=*−1.5018 *P=*0.9994	*T=*−0.6679 *P=*1.000	*T=*6.1286 *P=*0.2641	*T=*−5.5232 *P=*0.4011	1

Prairie lizards were subjected to current conditions or night-time warming of 2°C or 4°C and low, moderate or high prey availability. The table shows results from *post hoc* Tukey–Kramer pairwise analyses. Asterisks indicate a significant difference.

### Digestive passage time

Analysis of covariance revealed that the covariate of SVL significantly correlated with increased digestive passage time (ANCOVA covariate *P*<0.0054, *F*=8.186). Digestive passage time was significantly influenced by both night-time warming (ANCOVA *P*<0.001, *F*=226.3) and prey availability (ANCOVA *P*=0.001, *F*=7.7; Fig. 1B). There was a significant interaction between night-time warming and prey availability (ANCOVA *P*=0.0374, *F*=2.7). Specifically, when food availability was low, lizards experiencing current temperature conditions passed food slower than lizards experiencing 2°C night-time warming under both moderate and high prey conditions but not low prey conditions (Table 2). Additionally, digestive passage time was significantly slower under current conditions and low prey compared with all prey conditions under 4°C night-time warming.

**Table 2.** Night-time warming and prey availability showed an interaction effect with digestive passage time in prairie lizards (*S. consobrinus*)

Food availability	Temperature treatment	Low food			Moderate food			High food		
		Current	+2°C	+4°C	Current	+2°C	+4°C	Current	+2°C	+4°C
Low	Current	1								
	+2°C	*T*=26.7937 *P*=0.1894	1							
	+4°C	*T*=39.2154 *P*=0.0022*	*T*=−12.4217 *P*=0.9332	1						
Moderate	Current	*T*=−39.7504 *P*=0.0018*	*T=*12.9567 *P=*0.9162	*T=*0.5350 *P=*1.000	1					
	+2°C	*T*=42.8721 *P*=0.0004*	*T=*16.0784 *P=*0.7487	*T=*3.6567 *P=*1.000	*T=*3.1217 *P=*1.000	1				
	+4°C	*T*=39.0222 *P*=0.0033*	*T=*12.2285 *P=*0.9549	*T=*−0.1932 *P=*1.000	*T=*−0.7282 *P=*1.000	*T=*3.8499 *P=*1.000	1			
High	Current	*T*=−36.6789 *P*=0.0054*	*T=*−9.8853 *P=*0.9826	*T=*2.5365 *P=*1.000	*T=*3.0715 *P=*1.000	*T=*6.1932 *P=*0.9983	*T=*2.3432 *P=*1.000	1		
	+2°C	*T*=−39.2604 *P*=0.0021*	*T*=−12.4667 *P*=0.9318	*T=*−0.0450 *P*=1.000	*T=*0.4900 *P=*1.000	*T=*3.6117 *P=*1.000	*T=*−0.2382 *P=*1.000	*T=*2.5815 *P=*1.000	1	
	+4°C	*T=*−45.5694 *P=*0.0002*	*T*=−18.7757 *P*=0.5889	*T=*−6.3540 *P=*0.9983	*T=*−5.819 *P=*0.9991	*T=*−2.6973 *P=*1.000	*T=*−6.5472 *P=*0.9983	*T=*2.5815 *P=*1.000	*T=*−6.3090 *P=*0.9984	1

Prairie lizards were subjected to current conditions or night-time warming of 2°C or 4°C and low, moderate or high prey availability. The table shows results from *post hoc* Tukey–Kramer analyses. Asterisks indicate a significant difference.

### Fecal and urate production

**Fig. 2.**
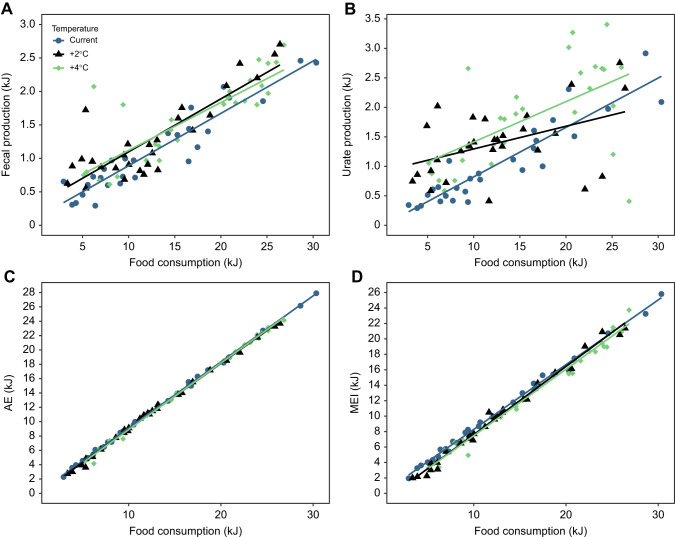
**The relationship between food consumption and fecal and urate production, assimilated energy (AE) and MEI in prairie lizards (*S. consobrinus*) subjected to night-time warming.** Night-time warming significantly influenced fecal (A) and urate (B) production in prairie lizards, where lizards experiencing warmer night-time temperatures produced significantly more feces than those under current conditions. However, MEI and AE were comparable across temperature treatments, as food consumption is a primary driver of the calculated budgets.

Energy (kJ) consumed by lizards significantly influenced fecal production (ANCOVA covariate *P*<0.0001, *F*=163.9), where lizards consuming more food produced more feces (Fig. 2A). Slopes of the relationships between consumption (covariate) and fecal production were homogeneous among treatment groups. Night-time warming significantly influenced fecal production (ANCOVA *P*=0.0006, *F*=6.4), while prey availability treatment did not. Specifically, lizards under current temperature conditions produced lower amounts of feces by 23.57% than lizards under 2°C night-time warming (Tukey *T*=−2.435, *P*=0.0448) and by 18.32% than lizards under 4°C night-time warming (Tukey *P=*0.0292), indicating higher assimilation per gram of food under current conditions (Fig. 2A).

Energy (kJ) consumed by lizards significantly influenced urate production (ANCOVA covariate *P*<0.0001, *F*=58.9), where lizards consuming more food produced more urates (Fig. 2B). Slopes of the relationships between consumption (covariate) and urate production were homogeneous among treatment groups. Night-time warming also significantly influenced urate production (ANCOVA *P*<0.0001, *F*=8.8), while prey availability treatment did not. Specifically, lizards under current temperature conditions produced lower amounts of urate by 38.38% than lizards under 2°C night-time warming (Tukey *T*=−3.603, *P*=0.0016; Fig. 2B), with lizards under 2°C and 4°C night-time warming producing comparable amounts of urate.

### Assimilated energy

Energy consumed (kJ) significantly influenced AE (ANCOVA covariate *P<*0.0001, *F*=2447.8; Fig. 2C), where lizards consuming more food had higher AE. Slopes of the relationships between consumption (covariate) and urate production were homogeneous among treatment groups. Assimilated energy was not influenced by night-time warming or prey availability treatment. There were no significant interactions.

### Metabolizable energy intake

Energy (kJ) consumed influenced MEI (ANCOVA covariate *P*<0.0001, *F*=1735.5), where lizards consuming more food had higher MEI. Night-time warming (ANCOVA *P*=0.0002, *F*=7.7) significantly influenced MEI, while prey availability treatment did not (Fig. 1D). However, there was a significant interaction between night-time warming and prey availability (ANCOVA *P=*0.01667, *F*=3.3; Fig. 1D). Specifically, lizards experiencing moderate prey availability under 2°C night-time warming had lower MEI by 7.76% than lizards experiencing low prey, and 9.32% than lizards experiencing moderate prey under current conditions (Table 3). Additionally, lizards experiencing moderate prey had higher MEI by 6.54% under 4°C night-time warming compared to 2°C night-time warming.

**Table 3.** Night-time warming and prey availability showed an interaction effect with metabolizable energy intake in prairie lizards (*S. consobrinus*)

Food availability	Temperature treatment	Low food			Moderate food			High food		
		Current	+2°C	+4°C	Current	+2°C	+4°C	Current	+2°C	+4°C
Low	Current	1								
	+2°C	*T*=−1.7070 *P*=0.7403	1							
	+4°C	*T*=−3.0973 *P*=0.0645	*T*=1.1303 *P*=0.9676	1						
Moderate	Current	*T*=0.7512 *P*=0.9978	*T=*−2.5750 *P=*0.2148	*T=*−4.0818 *P=*0.0034*	1					
	+2°C	*T*=−3.3042 *P*=0.0370*	*T=*−1.5999 *P=*0.8019	*T=*−0.4713 *P=*0.9999	*T=*−4.5259 *P=*0.0007*	1				
	+4°C	*T* =−0.0701 *P*=1.000	*T=*−1.7115 *P=*0.7376	*T=*2.9523 *P=*0.0928	*T=*−0.8462 *P=*0.9949	*T=*−3.6905 *P=*0.0119*	1			
High	Current	*T*=0.3067 *P*=1.0000	*T=*−1.3922 *P=*0.8973	*T=*−2.4785 *P=*0.2592	*T=*1.0755 *P=*0.9760	*T=*−3.4037 *P=*0.0280*	*T=*0.2909 *P=*1.0000	1		
	+2°C	*T*=−1.6616 *P*=0.7673	*T*=0.0478 *P*=1.0000	*T=*1.0429 *P*=0.9802	*T=*−2.6244 *P=*0.1942	*T=*−1.7149 *P=*0.7356	*T=*−1.9079 *P=*0.6106	*T=*−1.6979 *P=*0.7458	1	
	+4°C	*T=*−1.6351 *P=*0.7824	*T*=−0.1511 *P*=1.0000	*T=*−1.3599 *P=*0.9090	*T=*−2.6166 *P=*0.1974	*T=*−1.9697 *P=*0.5688	*T=*1.7342 *P=*0.7237	*T=*−1.4191 *P=*0.8870	*T=*−0.2168 *P=*1.0000	1

Prairie lizards were subjected to current conditions or night-time warming of 2°C or 4°C and low, moderate or high prey availability. The table shows results from *post hoc* Tukey–Kramer analyses. Asterisks indicate a significant difference.

## DISCUSSION

Our study showed that warming night-time temperatures and prey availability interacted to influence digestive physiology and energy budgets in *Sceloporus consobrinus*. Many studies emphasize the singular effects of temperature change or prey availability on animals (e.g. Yang et al., 2005; Barton and Yvon-Durocher, 2019; Thoral et al., 2021). However, the current study exemplifies how both temperature and prey abundance can have interactive effects on food consumption, digestive passage time and metabolizable energy. Specifically, our study examined the effect of prolonged exposure to high, moderate or low prey availability on digestive physiology, as opposed to just the effect of variable food consumption rates. Climate studies often focus on daytime temperatures or thermal extremes (Speights et al., 2017), but ectothermic animals that behaviorally thermoregulate during the day may experience the most drastic changes in *T*_b_ at night. Therefore, the effect of increasing night-time temperatures resulting in different energy budgets (MEI) may be of great concern. The results presented here suggest that a 2°C increase in night-time temperature maintained or increased rates of voluntary food consumption in lizards. However, a 4°C increase in night-time temperature had varying effects on how much lizards would consume, depending on prey availability. More specifically, we found that a 4°C increase in night-time temperature resulted in lizards voluntarily consuming less food when prey abundance was high when compared with cooler temperature scenarios. When adjusting for the effect of how much lizards ate, MEI budget was consistently higher under current thermal conditions in contrast to both a 2°C and 4°C increase in night-time temperature. Additionally, the effects of night-time warming were variable and non-linear across prey treatments. Therefore, night-time temperature is likely important for regulating energetics and digestive physiology in lizards, having significant interactive effects with prey availability.

As expected, lizards that consumed more food exhibited increased MEI and AE, as well as increased energy excreted in feces and urates. However, when adjusting for the influence of food consumption, prey availability and night-time temperature had interactive and non-linear effects on MEI, whereas AE remained consistent. While both fecal and urate production were highest for lizards under 2°C night-time warming and lowest for lizards under current conditions, the effect of night-time warming was most pronounced on urate production. Therefore, lizards were excreting significantly more energy as uric acid when night-time temperatures warmed, resulting in lower MEI. Such changes in MEI can be consequential for individual fitness, with direct effects on energy available for maintenance, growth, storage and reproduction. Using the experimentally derived equations previously described for metabolic rates during scotophase and photophase for *Sceloporus* lizards in the summer (Angilletta, 2001a), a 7 g lizard with high prey conditions under current temperatures (106.32 kJ MEI based on the current study) would experience a 39.76% and 63.09% decrease in kilojoules of energy in the budget during the reproductive season (March to mid-July) when warming by 2°C and 4°C at night, respectively.

Many thermal performance curves are based on measurements at stable temperatures (Huey and Stevenson, 1979; Pinch and Claussen, 2003; Telemeco, 2014). However, it is likely that cycling temperature regimes influence physiology differently from chronic exposure to stable temperatures (Litmer and Beaupre, 2024). The variable rates in digestive physiology observed here among daily temperature cycles suggest that warming night-time temperatures alone can influence performance and generate unique performance curves. In the current study, the temperature cycle yielding the highest rates of food consumption was the 2°C night-time warming treatment. Night-time temperature profiles may offer unique insight into performance in nature and play a critical role in regulating physiological processes. However, it is also plausible that processing rates may vary with temperature and prey availability. Additionally, considering the variation among physiological processes in relation to temperature cycles may be important, as food consumption followed a different pattern by peaking at 2°C warming in comparison to all other variables measured. Food consumption was voluntary and incorporates a behavioral choice by lizards, whereas other physiological processes here do not, which may in part contribute to the variation observed.

Digestive passage time interacted with both food availability and night-time temperature and was influenced by food consumption when lizards experienced current temperatures, with slower passage under the low prey treatment. As temperatures warmed at night, the effect of low prey on passage time became less prominent. Digestive processes are known to experience diminishing returns as food is processed over time in the gut and intestinal track (Levy et al., 2017). However, if animals are continuously refilling the gut when space is available, the digestive system is always engaged and, in part, operating at relatively high efficiency. One hypothesis for the change in passage time observed with night-time warming is lizards experiencing a decreased efficiency of digestive processes more frequently, and slower digestion, when food is low. The findings here are similar to those of a previous study, indicating that passage time is slower when lizards are fed restricted diets (Waldschmidt et al., 1986). Explicit studies need to be conducted to determine why passage time is slower when lizards consume lower amounts of food under cooler night-time temperatures. However, it is likely that the combined effects of low food and temperature interact to result in slow passage times.

Changes in night-time temperature have been found to influence other organismal processes than those studied here, with different effects observed among taxa. In side-blotched lizards (*Uta stansburiana*), warming night-time temperatures increased reproductive output, decreased the duration of the reproductive cycle and increased hatchling mass (Clarke and Zani, 2012). However, in common lizards (*Zootoca vivipara*), warming night-time temperatures decreased reproductive output and success (Brusch et al., 2023). Warming night-time temperatures also increased ectoparasitic infestation and altered allocation of resources towards growing in length instead of mass in common lizards (Rutschmann et al., 2021). The effects of night-time temperatures have also been studied in insects. Specifically, in English grain aphids (*Sitobion avenae*), warming night-time temperatures on warm days reduced survival and adult performance, and were predicted to have detrimental effects on the intrinsic rate of population growth (Zhao et al., 2014). In other insects, warming night-time temperatures are predicted to increase rates of development and allow insects to live closer to their thermal optimum (Speights et al., 2017). However, additional constraints may exist for insects, such as light availability and circadian rhythm. Therefore, the findings of the current study in conjunction with others indicate that warming night-time temperatures may increase ectotherm performance in some instances, but not always.

Previous research has examined food consumption in relation to daily temperature and activity (McCue, 2004). It was predicted that longer activity at warm *T*_b_ for lizards may not directly influence food consumption but may quicken digestion (Levy et al., 2017). However, when considering night-time temperatures in the current study, it was found that even if animals have slower digestion (such as in our low prey and current condition treatment), they may consume comparable amounts of food to lizards experiencing quicker digestion (such as in our low prey and +4°C condition treatment). When considering only stable temperatures, studies indicate that digestive performance follows a standard performance curve and increases with temperature until hitting an optimum (Angilletta, 2001a,b; Ojanguren et al., 2001), and the peak is higher if prey availability increases (Waldschmidt et al., 1986; Jonsson et al., 2001). Other studies using daily cycling temperature regimes have found similar results, where yolk absorption, growth rate, duration of larval stage and swimming kinematics occur at lower efficiency with reduced prey abundance despite temperature changes (e.g. Vidal et al., 2002; Mc Donnell et al., 2014; Allan et al., 2022). However, when using cycles in the current study, performance did not always increase proportionally to prey availability. Instead, we found non-linear interactions among night-time warming and prey availability to affect physiology. Some studies in natural systems have found similar interactions between temperature and prey affecting performance. For example, in whitespotted congers (*Conger myriaster*), individual growth rate was more sensitive to temperature when prey availability was low (Mu et al., 2022), and similar effects have been observed in dynamic energy budget modeling of other ectotherms (Beaupre, 2002). White crappies (*Poxmoxis annularis*) in reservoirs fed below physiological capacity despite high prey availability at moderate temperatures, but increased consumption when temperatures warmed in ways that were unreproducible under stable temperatures in the lab (Michaletz et al., 2012). Therefore, including temperature cycles in lab studies may better exemplify complex interactions in nature.

Evidence exists suggesting that night-time temperatures may be increasing more quickly than daytime temperatures (Vose et al., 2005; Davy et al., 2017), which is not commonly considered in organismal research on temperature change (e.g. Paaijmans et al., 2013; Verberk et al., 2016; Pontes-da-Silva et al., 2018). Meanwhile, many ectotherms face greater challenges with thermoregulating at night, due to the homogeneity of the thermal landscape. The current study is limited by assuming that animals will behaviorally thermoregulate to their average *T*_b_ daily. However, it is possible that variation in thermoregulation during the day is important as well, or animals will face difficulties in the future with daily thermoregulation. Furthermore, this study examined only three prey availability scenarios, with the intention of investigating the effects of consistent prey conditions rather than simply focusing on food consumption. It is possible that prey could vary in different ways from those considered here, which could result in different outcomes. The interactive and non-linear effects of increasing night-time temperature and altering prey abundance on digestive physiology may be prevalent among other taxa.

The abiotic and biotic environment can interact to influence bioenergetics, and such interactions can be unpredictable and non-linear. Prey availability can change in response to factors aside from climate change, including intraspecific and interspecific competition (Anderson, 2001). Alternatively, shifts in animal activity associated with temperature changes could alter foraging behaviors and predator–prey interactions, with cascading effects on physiology. The findings here could be expanded upon by considering how population ecology varies in response to both environmental and internal processes. Additionally, a thermal optimum for night-time temperature may exist and differ from the daily thermal optimum, influencing physiological processes. Findings of significant interactions between night-time warming and prey availability have implications not only for climate change and understanding how organisms interact with their environment but also for population-level processes. Specifically, the nuanced effect of daily temperature cycles and how concurrent environmental change can elicit variable effects warrants additional research. Overall, environmental changes can have prominent interactive and unpredictable effects on organismal physiology, which may pose long-term consequences for fitness or life history.
